# Magnetically Aligned Nanorods in Alginate Capsules (MANiACs): Soft Matter Tumbling Robots for Manipulation and Drug Delivery

**DOI:** 10.3390/mi10040230

**Published:** 2019-03-31

**Authors:** Lamar O. Mair, Sagar Chowdhury, Genaro A. Paredes-Juarez, Maria Guix, Chenghao Bi, Benjamin Johnson, Bradley W. English, Sahar Jafari, James Baker-McKee, Jamelle Watson-Daniels, Olivia Hale, Pavel Stepanov, Danica Sun, Zachary Baker, Chad Ropp, Shailesh B. Raval, Dian R. Arifin, Jeff W.M. Bulte, Irving N. Weinberg, Emily E. Evans, David J. Cappelleri

**Affiliations:** 1 Weinberg Medical Physics, Inc., North Bethesda, MD 20852, USA; sagar353@gmail.com (S.C.); brad.w.english@gmail.com (B.W.E.); sahar.jafari2011@gmail.com (S.J.); jbakermckee@gmail.com (J.B.-M.); jamelle.wd@gmail.com (J.W.-D.); oliviahale333@gmail.com (O.H.); pavstepan@gmail.com (P.S.); danica.sun5@gmail.com (D.S.); zgbaker54@gmail.com (Z.B.); chadropp@gmail.com (C.R.); rshailesh8504@gmail.com (S.B.R.); inweinberg@gmail.com (I.N.W.); 2 Multi-Scale Robotics and Automation Lab, School of Mechanical Engineering, Purdue University, West Lafayette, IN 47907, USA; maria.guix@gmail.com (M.G.); bi10@purdue.edu (C.B.); john1360@purdue.edu (B.J.); 3 Russel H. Morgan Department of Radiology, Division of Magnetic Resonance Research, The Johns Hopkins University School of Medicine, Baltimore, MD 21205, USA; genaro.paredes@gmail.com (G.A.P.-J.); darifin@mri.jhu.edu (D.R.A.); jwmbulte@mri.jhu.edu (J.W.M.B.); 4 Cellular Imaging Section and Vascular Biology Program, Institute for Cell Engineering, The Johns Hopkins University School of Medicine, Baltimore, MD 21205, USA; 5 Department of Oncology, The Johns Hopkins University School of Medicine, Baltimore, MD 21205, USA; 6 Department of Biomedical Engineering, The Johns Hopkins University School of Medicine, Baltimore, MD 21205, USA; 7 Department of Chemical & Biomolecular Engineering, The Johns Hopkins School of Engineering, Baltimore, MD 21218, USA; 8 Department of Physics, Elon University, Elon, NC 27244, USA; eevans23@elon.edu

**Keywords:** alginate capsules, magnetic nanorods, magnetic microrobots, tumbling robots, surface walkers, rotating magnetic fields, micromanipulation

## Abstract

Soft, untethered microrobots composed of biocompatible materials for completing micromanipulation and drug delivery tasks in lab-on-a-chip and medical scenarios are currently being developed. Alginate holds significant potential in medical microrobotics due to its biocompatibility, biodegradability, and drug encapsulation capabilities. Here, we describe the synthesis of MANiACs—Magnetically Aligned Nanorods in Alginate Capsules—for use as untethered microrobotic surface tumblers, demonstrating magnetically guided lateral tumbling via rotating magnetic fields. MANiAC translation is demonstrated on tissue surfaces as well as inclined slopes. These alginate microrobots are capable of manipulating objects over millimeter-scale distances. Finally, we demonstrate payload release capabilities of MANiACs during translational tumbling motion.

## 1. Introduction

Untethered milli- and microscale robotics have reached impressive levels of complexity and are now capable of performing a several tasks in vitro and in vivo [1,2,3,4,5,6,7,8,9]. A wide variety of materials have been explored, with varying levels of attention to their toxicity and decomposition properties. While most of these milli- and microscale swimming and tumbling robotic devices rely primarily on hard metallic compositions, a growing interest in soft, polymer-based manipulation devices exists due to their applicability in medicine and surgery. Alginate is a naturally occurring polymer derived from brown algae which has been extensively used in drug and cell delivery systems [10]. Owing to its biodegradability, non-toxicity, non-immunogenicity, ease of processing, and low cost, alginate has been broadly employed across a variety of medical devices [11]. Capsules of alginate ranging in size from tens of nanometers [12] to several millimeters [13] can be implanted in humans with minimal side effects [14]. As a result, alginate capsules incorporating a broad array of payloads have been deployed for a variety of purposes, including drug delivery, cell encapsulation, bone regeneration, wound healing, and regenerative medicine [15]. Clinical trials implanting these capsules have aimed to use islet cells embedded in alginate capsules as cellular factories for the secretion of regulating molecules such as insulin [16,17,18]. Imbuing alginate capsules with a means of propulsion and position control has the potential to expand their applicability as medical milli- and microrobots. Recently, alginate-based microrobots have been used as controlled steering manipulators [19] and cell culture delivery devices [20]. 

The field of milli-, micro-, and nanoscale robotics in medicine aims to apply physical and chemical techniques for steering motile devices for deployment in the body [1]. Techniques for imparting motility generally rely on chemical gradients/catalysis [21], magnetic actuation [22], acoustic propulsion [23,24,25,26], biological propulsion [27,28], or combinations of such propulsion mechanisms [29]. Excellent reviews of these various methods have appeared elsewhere [1,2,3]. The biocompatibility and penetration depth of magnetic fields inside the body makes magnetic methods of propulsion appealing for device guidance and targeted drug delivery. Significant progress has also been made in moving small, magnetically operated robots towards clinical use [30]. Methods of magnetically guiding objects through and along biological barriers have often relied on magnetic field gradients either for pulling the particles towards the source of the magnetic field [31,32,33], or for pushing particles away from the source of the magnetic field [34,35]. Increasingly, rotating magnetic fields have been used for actuating particles via the process of surface tumbling [4,8,36,37,38,39,40]. Surface tumbling uses rotating magnetic fields to induce rotation of paramagnetic or ferromagnetic particles near a surface. Some of the rotationally manipulated surface tumblers demonstrate true surface rolling via contact with the surface [8,41,42]. Others rely on a fluid mediated interface with the surface, where the rotation of a body near the surface boundary results in differential drag on the particle surface. The no-slip condition at the solid-fluid boundary dictates that segments of the object near the surface experience different drag conditions compared with the segments of the object farther away from the surface, which breaks the symmetry of motion, a requirement for translation at low Reynolds numbers [43]. Surface tumbling objects ranging from hundreds of nanometers to hundreds of micrometers have been developed and applied to various in vitro tasks, including generating macroscopic flows [43], picking up and dropping off cargos [44], breaking apart blood clots [45], manipulating individual cells [39], disrupting biofilms [46], moving particles opposite to a macroscopic flow [47], harvesting protein crystals [48], and generating vortices capable of carrying other objects [49]. Micro- and nanoscale magnetic surface tumblers have been manipulated at translational velocities >0.3 body lengths/rotation [50] and 1 kHz rotational frequencies [51]. Such magnetic tumblers have, for the most part, been relegated to manipulation on smooth, flat, rigid surfaces. However, early work on rolling microrobots proposed surface tumblers for navigation within and atop tissue surfaces [4,41,42,52], and recent work using microtumbling robots has demonstrated the ability to move lithographically manufactured structures on wet and dry surfaces, as well as across complex surfaces and inclines [8]. 

Alginate capsules loaded with magnetic particles have been fabricated [53,54,55,56,57,58] and implemented as magnetic resonance imaging contrast agents [59,60,61,62,63], magnetic hyperthermia-triggered drug release agents [64], and magnetically actuated microrobots [19]. The ability to steer an alginate capsule carrying a payload may prove beneficial for spatially defined payload delivery [20]. A single platform capable of overcoming all functional challenges, including: (1) traversing rough or biological surfaces, (2) climbing inclines, (3) manipulating other exterior objects, and (4) delivering molecular or therapeutic payloads, has not yet been demonstrated using alginate-based microrobots. Here we demonstrate a microrobotic platform capable of overcoming all four previously mentioned functional challenges. We synthesize the first magnetically aligned nanorods in alginate capsules (MANiACs), imbuing alginate capsules with fixed magnetic dipoles via aligned nanorods. Due to their high magnetization, aligned magnetic nanorods in alginate capsules allow capsules to be rotated with small magnetic fields using relatively low loading volume fractions. Additionally, because nanorods are ferromagnetic, the torque applied to the nanorods is conferred directly to the alginate capsule and not to rotating magnetic domains only, as may be possible with paramagnetic particle loading. The magnetic ordering of the capsule is fixed by the aligned rods, which are fixed in the alginate matrix. We demonstrate rotationally guided translation of MANiACs traveling on glass surfaces, biological tissue surfaces (rat intestine), and inclined surfaces over centimeter-scale distances. We demonstrate their ability to manipulate other millimeter-scale objects and demonstrate combined steering and release of a model molecular payload. Increasingly, millimeter-scale robotics are being designed to accomplish various tasks in the gastrointestinal tract [65,66]. As alginate is a commonly used food additive suitable for ingestion, alginate-based microrobotic platforms such as MANiACs may hold significant potential for operating as magnetically guided robots for gastrointestinal interventions.

## 2. Materials and Methods

### 2.1. Nanorod Synthesis

We synthesized multi-segmented Au-Ni-Au magnetic nanorods using standard template guided electroplating techniques [67,68,69]. Whatman Anodisc 13 anodized aluminum oxide (AAO) membranes with nominal pore diameters of 200 nm were sealed on one side with 600 nm of thermally evaporated silver. Nanorods were grown via DC electrodeposition into the pores of the AAO using gold and nickel-plating solutions (Technic Inc., Cranston, Rhode Island, Orotemp 24 and 1.1 M NiSO_4_·6H_2_O, 0.2 M NiCl_2_·6H_2_O, and 0.75 M H_3_BO_3_, respectively). Etching the evaporated silver layer and AAO membrane using nitric acid and 1 M NaOH, respectively, released nanorods from their templates. We rinsed nanorods repeatedly by magnetic separation and dispersed them in 10 mM HEPES (4-(2-hydroxyethyl)-1-piperazineethanesulfonic acid) buffer prior to mixing with alginate.

### 2.2. MANiAC Synthesis

**Figure 1:**
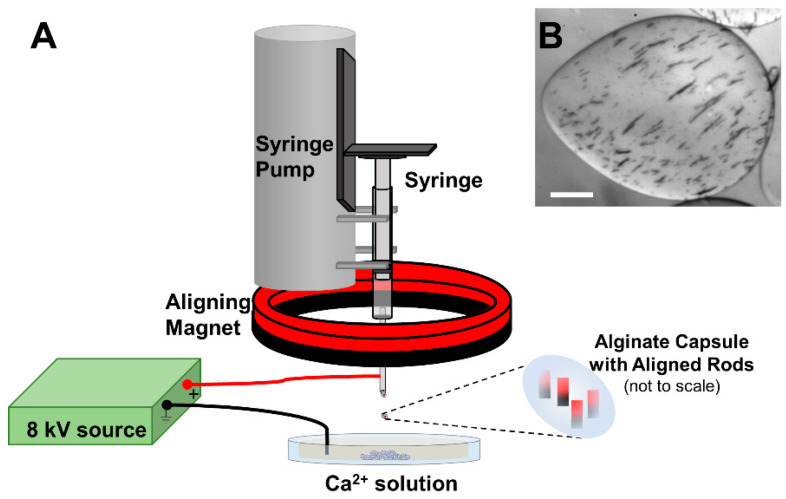
MANiAC synthesis. (**A**) A syringe pump pushes the mixture of magnetic nanorods and alginate through an aligning magnet prior to reaching the blunted needle tip. A high voltage source supplies ~8 kV between the Ca^2+^ solution and the syringe generates a dropwise extraction of capsules embedded with aligned nanorods. (**B**) Optical image of aligned nanorods in alginate capsule. Scale bar = 100 µm.

Alginate synthesis techniques have been reviewed and details have appeared elsewhere [70,71]. Briefly, using ultrapure alginate (Pronova UP LVG, NovaMatrix cat. #4200001, Sandvika, Norway) we mixed filtered liquid alginate with magnetic nanorods and loaded the mixture into a syringe. A syringe pump pushed the alginate/nanorod mixture out at 200 µL/min through a blunted 24G syringe tip. We formed alginate capsules using a high voltage droplet generation method, applying an ~8 kV bias between the syringe tip and a bath of 100 mM CaCl_2_ in 10 mM HEPES buffer solution, positioned approximately 10 cm below the syringe tip. Nanorods were aligned in the capsule by passing the alginate/nanorod solution through an axially magnetized NdFeB ring magnet (K&J Magnetics, magnet RZ0Y0X03, Pipersville, PA, USA) prior to extraction of the MANiACs from the syringe tip (Figure 1A). We allowed the MANiACs to settle to the bottom of a conical tube for 5 minutes and later removed calcium chloride solution, rinsing the capsules three times with 25 mL of Krebs-Ringer-HEPES buffer (containing 2.5 mM CaCl_2_). The MANiAC dimensions ranged from 600 µm to 1200 µm.

### 2.3. Magnetic Manipulation of Capsules

We manipulated the MANiACs in DI water or 10 mM phosphate buffered saline (PBS, pH = 7.4) using uniform magnetic fields between 5 mT and 20 mT and rotation frequencies between 0.5 and 2 Hz supplied by a coil array (MFG-100, MagnebotiX AG, Zurich, Switzerland) [72]. We captured videos at 20 or 30 frames per second using an overhead charge-coupled device (CCD) camera (Basler puA1600-60uc, Basler AG, Ahrensburg, Germany, baslerweb.com) along with microscope lenses (Edmund VZM 450i, Edmund Optics, Barrington, NJ, USA, www.edmundoptics.com).

### 2.4. Intestine Preparation for Tissue Surface Tumbling

We prepared the small intestine of Sprague Dawley rats (BioreclamationIVT, Hicksville, NY, USA) by slicing open the intestine lengthwise and laying the tissue flat on a glass slide with the exterior surface of the intestine placed down in contact with a glass coverslip. We placed a droplet containing the MANiACs on the glass slide beside the intestine and applied a rotating magnetic field. In response to the rotating magnetic field, the MANiACs rotated in phase with the rotating field and translated onto and over the intestine. We measured translational velocity on the glass and the rat intestine using Video SpotTracker (version 8.11, Computer Integrated Systems for Microscopy and Manipulation (CISMM), The University of North Carolina at Chapel Hill, Chapel Hill, NC, USA) [73]. 

### 2.5. SU-8 Microstructures Fabrication

We synthesized polymeric T-shaped hollow microstructures using standard photolithographic processes. Following the standard recommended protocol for SU-8 50 (Microchem Inc., Westborough, MA, USA), we spin-coated SU-8 50 onto a silicon wafer at 1000 rpm for 30 s, then soft baked the wafer using two consecutive treatments (10 min at 65 °C and 30 min at 95 °C). Using a mask aligner (350–400 nm light, Suss MA 6 Mask Aligner, SUSS MicroTec, Garching, Germany), we exposed the sample for 72 s, followed by a post-bake of 1 min at 65° C. We used SU-8 developer to remove the non-polymerized photoresist and recovered the resulting T-shaped microstructures with horizontal and vertical lines with dimensions 2.0 mm × 1.75 mm and 0.75 mm × 1.75 mm, respectively. We placed the T-shaped structures in the sample region, adding the MANiACs to the sample region using a micropipette. In a rotating magnetic field, the MANiACs translated and, when in contact with a T-shaped structure, pushed the structure across the cover slip.

### 2.6. Loading and Release Studies

We loaded the MANiACs with brilliant green (BG) dye via 24-hour incubation in 100 mM BG solution, followed by centrifugal separation and rinsing with ultrapure 18 MΩ-cm water. The MANiACs have permeable shells, which allowed the BG loading by diffusion through the shell layer and into the capsule volume. After rinsing, we dispersed the MANiACs in PBS for osmotic release studies, removing 10 µL aliquots for spectroscopic quantification of BG content at 625 nm. To demonstrate combined surface tumbling and BG release, we placed a transparent petri dish containing a single MANiAC onto a backlit sheet of grid paper, manipulating the MANiAC with a rotating magnetic field while observing BG release. 

## 3. Results and Discussion

### 3.1. Synthesis and Actuation of Magnetically Aligned Nanorod Alginate Capsules (MANiACs)

**Figure 2:**
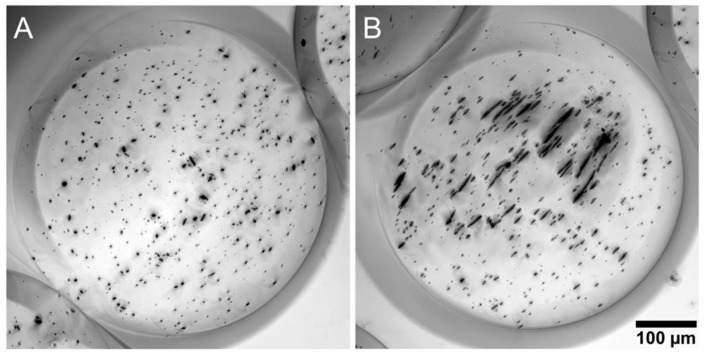
Minimum intensity projections taken from stacks of alginate capsules with (**A**) unaligned nanorods and (**B**) aligned nanorods.

Unlike previous magnetic alginate capsules containing superparamagnetic nanomaterials, incorporation of aligned ferromagnetic nanorods results in capsules with permanent magnetizations in the direction of the aligned rods. The MANiAC synthesis process differs from the standard electrostatic droplet generation method by the addition of a nanorod-aligning permanent magnet during the alginate capsule generation (Figure 1A). As the nanorod-loaded alginate mixture moves towards the blunted tip of the syringe, it passes through the ring-shaped aligning magnet that supplies an aligning magnetic field of ~0.3 T, creating capsules with well-aligned embedded nanorods (Figure 1B). When exposed to the aligning magnetic field, rods experience a magnetic torque *τ_m_* described as

(1) $$\tau_{m}=V_{m}\vec{M}\times \vec{B}=\pi r^{2}L\left|M_{r}\right|\;\left|B\right|\sin\theta$$

where $V_{m}$ is the volume of magnetic material, $\vec{M}$ is the magnetization of the rod, $\vec{B}$ is the magnetic field, $M_{r}$ is the remnant magnetization, *r* is the rod radius, *L* is the rod length, and θ is the lag angle between the nanorod and the applied magnetic field [74]. Here, we assume rod magnetization is along the length of the rod. This assumption is valid as long as the magnetic anisotropy energy [75] is large compared to the Zeeman energy density, or $0.25\mu_{0}M^{2}\gg MB$ for a high-aspect-ratio cylinder (AR > 10). This condition is satisfied in our nanorod system, in which *M* ~ 500,000 A/m and *B* ≤ 0.02 T. With numerical energy minimization, we are able to predict the magnetic torques on the nanorods independently of this assumption, and we find that under all experimental conditions the calculated torques are well within 1% of the approximation. From equation (1) we see that the maximum torque occurs when the applied magnetic field is perpendicular to the capsule’s magnetic moment. The remnant magnetization $M_{r}$ of nickel nanorods used in these experiments is ~5 × 10^5^ A/m, which is in accordance with other measurements of nanorods of the same composition [76]. Applying equation (1) to nanorods undergoing magnetic alignment, we find the maximum torque on a single nanorod to be ≈40 fN·m. The applied torque on the magnetic rods induces rotation of rods as they move through the ring-shaped magnet suspended in fluid phase alginate. The femtonewton estimate of torque is in accordance with other experiments involving forces and torques supplied to nanorods [33] and nanoparticles [77] of similar size in similar magnetic fields. Importantly, any mechanical disruption to the fluid droplet as it travels through air from the blunted tip of the syringe to the calcium chloride solution reservoir does not significantly disrupt magnetic nanorod alignment. The gelation process is initiated when the droplet contacts the calcium chloride solution. Upon gelation, the alginate crosslinks, becoming a non-Newtonian gel. The density of the alginate network forms a barrier of steric hindrance around the magnetic rods, functionally locking the nanorod orientation in place and coupling magnetic torque on nanorods to rotation of the entire MANiAC. To confirm that nanorod alignment is magnetically controlled as opposed to induced by hydrodynamic shear generated during synthesis, we synthesize a control sample of nanorod-containing alginate capsules generated without magnetic alignment (Figure 2). The control sample alginate capsules contain randomly oriented nanorods. Minimum intensity projections from images collected at various heights demonstrate alignment differences between unaligned nanorod control capsules (Figure 2A) and magnetically aligned nanorod capsules (Figure 2B). Additionally, the images indicate that nanorod alignment occurs throughout the capsule volume. Generally, capsules were elongated ellipsoids with an average length of 803 ± 49 µm and an average width of 444 ± 32 µm (n = 20 capsules). Capsule size is controlled by choice of synthesis parameters and apparatus which determine the size of the droplet at the tip of the syringe needle. As the droplet forms at the tip of the syringe needle, extraction from the tip occurs at a critical size that depends on alginate viscosity, extraction voltage field strength, and syringe needle gauge. Lower viscosity, higher extraction voltage field strengths, and smaller syringe needles will create smaller capsules. Nanorod density can be controlled by adjusting the density of rods in the alginate fluid phase. Prior to capsule formation, nanorods were thoroughly sonicated in buffer, and mixed in the alginate precursor by vortex. However once in the syringe, nanorod motion is controlled by diffusion, flow in the syringe, magnetic field supplied by the aligning magnet, and magnetic rod-rod interactions. As such, distribution of nanorods in the capsule is nonuniform.

### 3.2. Magnetic Surface Tumbling

In our experiments we manipulate MANiACs using rotating magnetic fields with negligible gradients across the region of interest. It should be noted that alginate capsules containing randomly oriented magnetic nanorods do not rotate in an applied rotating magnetic field, as their overall magnetic ordering is insufficient to couple with the weak magnetic fields used for manipulation. In a rotating magnetic field, capsules with randomly oriented nanorods have no translational velocity and their motion is guided by Brownian diffusion and gravity only. Uniform magnetic fields do not exert magnetic forces on the MANiACs, but they will exert torques. Magnetic nanorods transmit the magnetic torques to the alginate capsules, causing them to rotate. Since this rotation occurs near a boundary, the viscous fluid couples the rotational motion to translational motion. Additionally, the capsules experience intermittent direct contact with the surface, which we will discuss in subsequent sections. From equation (1) we see that the maximum torque occurs when the applied magnetic field is perpendicular to the capsule’s magnetic moment and we calculate the total torque on a single rod in the capsule as 1.3 × 10^−15^ N·m in the ~10 mT rotating fields applied during the experiments. For fluid mediated motion, the net force on the sphere in the plane of the floor is composed of two elements: the horizontal force induced by the rotation of the sphere near the floor ( $F_{x}^{r*}$ ), and the viscous drag force due to the resulting translation of the sphere near the boundary ( $F_{x}^{t*}$ ). Here, we will take the x axis as the axis of translation. In this low-Reynolds system, the net force on the sphere is zero and so these two forces are balanced:

(2) $$F_{x}=6\pi \eta r\left(vF_{x}^{t*}+r\omega F_{x}^{r*}\right)=0$$

Thus, the translational velocity, v , is given in terms of the rotational velocity, ω , as

(3) $$v=-\frac{F_{x}^{r*}}{F_{x}^{t*}}r\omega$$

**Figure 3:**
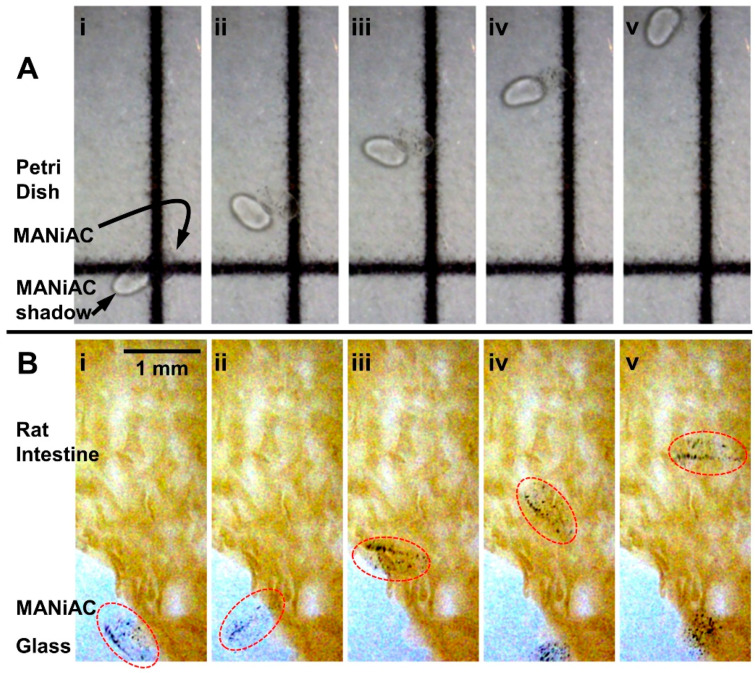
(**A**) MANiACs can be made to traverse straight lines. In a Petri dish over grid paper, a MANiAC is rotated at 0.5 Hz in a 20 mT rotating magnetic field, following the straight line of the grid paper. Images are extracted at 2.5 second increments. Note: MANiAC and MANiAC shadow are both observable in (A) due to the placement of the light source. The shadow appears slightly below and the left of the MANiAC itself. (**B**) The MANiAC starts translating on glass (**i**,**ii**), climbs onto the intestine surface (**iii**), and proceeds translating across the rat intestine surface (**iv**,**v**). Images are extracted at 1.5 s increments. Movies of (A) and (B) are included as electronic supplementary information (ESI movie S1 and S2).

**Figure 4:**
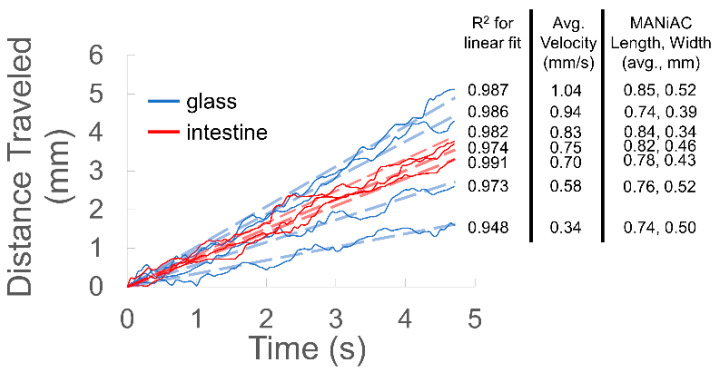
MANiAC velocity on glass and intestine surfaces. Linear fits of the capsule motion, average capsule velocity, and capsule size are also included.

Here, the coefficients $F_{x}^{r*}$ and $F_{x}^{t*}$ cannot be expressed in a closed form, but in general they depend on the sphere radius *r* and the separation from the floor, *h*. Several series expansions valid under a variety of conditions have been reported [78,79,80,81]. Notably, in an unbounded fluid ( h/r=∞ ) the coupling between rotation and translation, $F_{x}^{r*}$ , is zero and so rotation does not result in translation. However, the ratio $-F_{x}^{r*}/F_{x}^{t*}$ increases from zero as the sphere approaches the floor ( h/r→0) . In the limit of zero sphere-floor separation, the ratio approaches a limit of 0.25 and thus, the maximum translational velocity in this fluid-mediated mechanism is expected to be v=0.25 ωr , or one-quarter the rate expected from rolling without slipping [78]. In fluidic scenarios, rotating magnetic fields (<20 mT) are sufficient to induce MANiAC rotation and translation on glass, climbing onto rat intestine, and continuous translation on top of rat intestine (Figure 3). Changes in a surface from glass to tissue do not appreciably impact translational velocities (Figure 4). 

Translation efficiency can be calculated as measured translational velocity *v* divided by the maximum translational velocity for a capsule rolling in contact with the substrate, *v*_max_ = 2*πrω*. A capsule rolling in good contact with the substrate would exhibit an efficiency of 100%, while a particle which slips near the substrate would translate with a ≤25% efficiency, as described above. In the tracking data shown in Figure 4, capsules tend to rotate largely about their long axes, and so computed rolling efficiencies for the spheres range between 20% and 80%. Thus, it is evident that capsules tend to translate with a combination of sticking (100% efficiency) and slipping (maximum 25% efficiency) as they experience intermittent contact with the substrate. Indeed, longer particles tend to exhibit greater rolling efficiencies (% efficiency ∝ L, with R^2^ = 0.8352 omitting 1 outlier), which is consistent with the increased opportunity for contact afforded by a longer capsule. The strength of coupling between the MANiACs and nearby surfaces is determined by the bulk viscosity of the fluid, the geometric properties of the tumbler, proximity to the surface, capsule geometry, and rotational frequency. For a sphere near a surface, previous work has demonstrated that rotation and translation are coupled [37]. Capsules located close to the surface are more strongly coupled to the surface, and thus have higher coupling efficiency and move farther per rotation, with 100% coupling resulting in translation distances of one circumference per rotation, i.e., true rolling [41,42]. Tumbling over glass and the surface of rat intestines, the MANiACs move at translational velocities up to ~1 mm/s at 1 Hz (Figure 4). For the MANiACs with diameters 0.6–1.2 mm, true rolling would result in velocities ≈1.88–3.78 mm/s. Here, coupling results in translation efficiency between 20% and 80%, verifying that the MANiACs operate using a combination of direct contact rolling and fluid mediated tumbling. Despite the large number of reports describing surface tumbling nano-, micro-, and milliscale robots, very few have successfully tumbled on tissues [4,8]. 

Recent work on microrobots for targeting specific gastrointestinal tract regions have suggested the potential for highly localized delivery of payloads to diseased tissues. One application space for surface tumbling microrobots is shuttling cargo from place to place in vivo or in lab-on-a-chip and organ-on-a-chip devices. The ability to tumble across rough, soft tissue surfaces is of critical importance in these applications. To demonstrate this, we section excised intestine of Sprague Dawley rats and lay the tissue flat on a glass cover slip, with the inner surface of the intestine face up. The rat intestine presents a rough, elastic, biologically relevant surface for tumbling magnetic microrobots. We manipulate the MANiACs over the surface of rat intestine tissue (Figure 3) and demonstrate no appreciable decreases in translational velocities (Figure 4). In vivo, the intestine surface has a layer of mucus that protects the tissue from bacterial attack and regulates intestine function and transport. The dense concentration of mucins and resulting viscoelasticity of mucus is a well-known transport inhibitor for micro- and nanoscale particles [82]. Smaller, nanoscale surface tumblers may have trouble moving over such mucosal layers. However, the millimeter-scale MANiACs move over the intestine surfaces uninhibited due to their large size relative to the mucus interaction surface, as well as the large force applied to them compared with forces on smaller, nanoscale magnetic particles rolling over mucus membranes. 

Unlike previous magnetic microrobots capable of operating in both wet and dry environments [4,8,52], the MANiAC manipulation (in fields <20 mT) relies on the presence of a fluid. Torque supplied by the rotating magnetic field is insufficient to overcome electrostatic and van der Waals forces between the capsule and the glass surface under dry conditions. This is likely due to the relatively low magnetization of the MANiACs compared with previously fabricated microrobots capable of rolling on dry surfaces. 

### 3.3. Climbing Inclined Surfaces and Pushing Millimeter-Scale Structures

**Figure 5:**
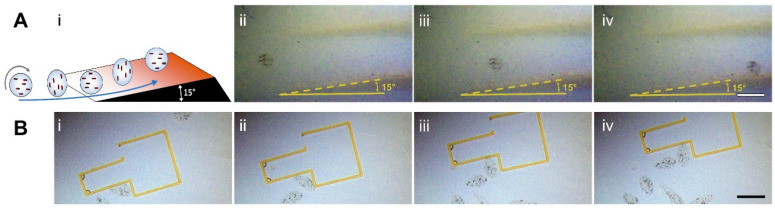
(**A**) Rotating MANiACs are able to climb 15° inclines. (**i**) Schematic depicting MANIACs rotation and incline climbing. (**ii**–**iv**) An alginate capsule climbing a 15° incline. Scale bar is 1 mm. (**B**) MANIACs are tumbling next to the corner of a hollow photoresist structure (**i**,**ii**), resulting in a successful manipulation by pushing the structure on a smooth glass surface (**iii**,**iv**). Scale bar =1 mm. For both (A) and (B) movies are included as electronic supplementary information, see ESI movies S3 and S4, respectively.

In addition to translating over tissue surfaces, the MANiACs can climb up 15° inclines (Figure 5A) and perform effective pushing of a millimeter-scale structure (Figure 5B). The ability to climb up inclined surfaces significantly increases a robot’s usefulness and adaptability for tasks on topographically complex surfaces. To be useful in three-dimensional lab/organ-on-chip environments and in vivo, the MANiACs will need to be able to crawl up inclined surfaces against the force of gravity. To demonstrate that ability, we place the MANiACs in solution at the base of a 3D printed 15° incline fabricated using a Form 1+ SLA 3D printer from Formlabs (Formlabs, Somerville, MA, USA, www.formlabs.com). Rotation at 1 Hz in a 10 mT magnetic field is sufficient to enable the MANiACs to climb the ramp (Figure 5A). Above 1 Hz, the 10 mT magnetic field is insufficient to keep the MANiAC phase-locked with the rotating magnetic field and phase-slipping occurs, disrupting climbing progress (results shown in ESI movie S2). Increased magnetic nanorod loading or increased magnetic field strength will allow for higher frequency phase-locking and improve the climbing speed. Increasing rotational frequency under phase-locked conditions may also increase the maximum incline angle the MANiACs can climb.

The fine manipulation of objects by magnetic robots at both the nanoscale [44] and microscale [48,83] has been demonstrated by taking advantage of the fluid microvortices generated by the robot rotation. Here, we manipulate objects with an overall size four times larger than our MANiACs (based on outer dimensions) by directly pushing the objects using the rotating MANiACs. Placing a T-shape microstructure with a length of 2.7 mm and width of 2.1 mm (at its widest dimension) in the vicinity of the alginate capsule surface tumblers, we show that capsules can push the structure across the surface with rotating magnetic fields <20 mT (Figure 5B and ESI movie S3). The photoresist structure is 486 µm tall, has a total volume of 0.14 mm^3^, and a total mass of 0.16 mg. The MANiACs have a mass of ~0.8 mg. The manipulation capabilities shown by the MANiACs suggests their potential application in microfluidic devices to shuttle collections of corralled cells for applications in cellular assembly.

### 3.4. Model Drug Loading, Release, and Combined Tumbling and Release

**Figure 6:**
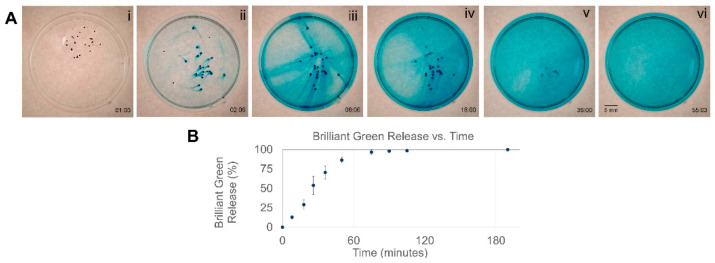
(**A**) Brilliant green dye (BG) release from the MANiACs over the course of 55 min. (**i**) Control experiment is run by evaluating the MANiAC release rates in presence of DI water, which are negligible. By adding phosphate buffered saline (PBS) (**ii**), BG release is triggered via osmosis. The MANiACs continue to release BG (**iii**,**iv**), and after 36 min many MANiACs are nearly clear (**v**). After 55 min nearly all loaded BG has been released (**vi**). (**B**) Spectroscopy measurements at 625 nm were done to quantify the drug release timeline. A movie is included in supplementary information (ESI movie 5).

**Figure 7:**
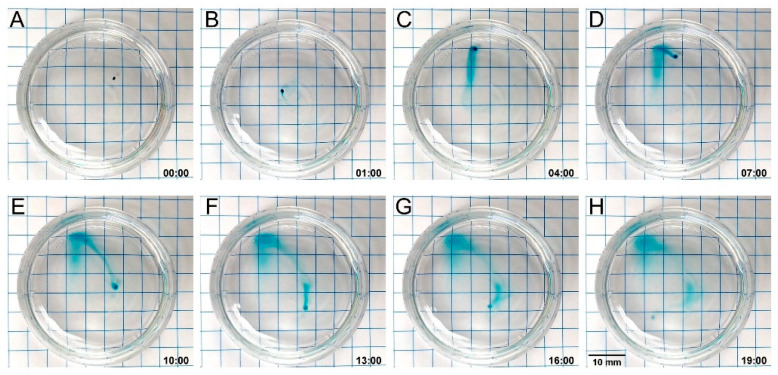
A single brilliant green-loaded MANiAC is placed in a petri dish (24 mm diam.) with rotating magnetic field-induced tumbling. Panel (**A**) is shown at time 0 min, panel (**B**) is shown at time 1 min, and subsequent panels (**B**–**H**) are spaced at 3 min increments. (A) The MANiAC is placed in DI water. (**B**) PBS is added to the petri dish and the MANiAC is manipulated to the left, followed by manipulation upwards (**C**). Manipulation is momentarily paused (**C**–**D**), allowing for a concentrated deposit of BG. Subsequent tumbling (**E**–**H**) completes the translation with nearly full release of BG (**H**). A video is included in supplementary information as ESI movie S6.

Alginate has a long history as a drug carrier, being extensively used as capsules for controlled release studies [84]. Translation via tumbling may serve as a method for transporting drug-loaded MANiACs to specific locations in vitro or in vivo. Here, loading the MANiACs with a model small molecule (brilliant green, BG, MW 475.6 g/mol), we characterize BG release in PBS and demonstrate combined tumbling translation and payload release. Figure 6A demonstrates the release of BG via time sequenced images, while Figure 6B shows BG release data acquired via spectroscopy (625 nm) from BG release experiments at various times. These experiments indicate that small molecular weight drugs (<1 kDa) are fully released from the MANiACs in approximately 1 hour. Focusing on the steering and release of a single capsule, Figure 7 depicts a time sequence in which a MANiAC releases the BG as we steer it in various directions in a petri dish. As shown in Figure 7, the MANiAC demonstrates an initial burst release of BG, followed by a tapering of the release rate. By pausing tumbling translation, we are able to differentially distribute amounts of BG at various locations across a surface. Thus, the MANiACs are controlled to deposit large molecular concentrations at given locations (Figure 7C,D,F,G). For in vitro applications such as lab- or organ-on-chip payload delivery, such depositions may allow for location-specific delivery of payloads at specific sites, allowing some regions of the chip to receive initial payload doses ahead of other regions.

Engineering manipulations of MANiACs with molecular payloads may enable payload concentrations profiles to be concentrated at specific locations or distributed more evenly throughout a given volume. Here, payload release is osmotic and occurs upon the addition of PBS to DI water. To improve upon release capabilities of these capsules, we anticipate magnetically triggerable payload release via radiofrequency heating of loaded nanorods. Future capsules may include more densely crosslinked outer shells or secondary coatings capable of decreasing or inhibiting osmotic transport and allowing for magnetically triggered payload release via hyperthermia induced by alternating magnetic fields [57].

Currently, the gastrointestinal tract is a promising tissue for micro- and millirobotic interventions [85]. Recent work has demonstrated location-specific delivery using template-synthesized micromaterials [86], and future work may seek to further control position and release of payloads, or guide cells to specific locations within the tract. While the safety of alginate capsules has been thoroughly investigated in animal tests and human trials, future work will aim to determine safety profiles for MANiACs in various tissue and organ systems. Followup work exploring biocompatibility for capsules containing various sizes of rods as well as various concentrations of rods will assist in understanding where and how these composite materials may be useful in vivo. 

## 4. Conclusions

We have successfully developed magnetically guidable surface tumbling millirobots, dubbed MANiACs, composed of magnetically aligned nanorods in alginate capsules. Guided by <20 mT rotating magnetic fields, MANiACs can traverse rough tissue surfaces, climb inclines, move nonmagnetic structures, and carry molecular payloads to specific locations in vitro. These are the first alginate capsules with an overall aligned magnetization orientation provided by incorporation of aligned ferromagnetic nanorods. Future incorporation of additional payloads, such as islet cells, drugs, biomarkers, nucleic acids, and ultrasound, magnetic resonance, or nuclear medicine imaging agents, may enable their use as minimally invasive, magnetically guided, site-specific theranostic devices.

## Supplementary Materials

The following are available online at https://www.mdpi.com/2072-666X/10/4/230/s1, Video S1: Single MANiAC tumbling in a Petri dish along a straight line; Video S2: MANiACs tumbling from glass to tissue surfaces; Video S3: MANiACs climbing up 15° lithographically produced incline; Video S4: MANiAC pushing lithographically generated structure composed of SU-8 photoresist; Video S5: Release of model drug (Brilliant Green dye) from stationary MANiACs; Video S6: Combined tumbling manipulation and model drug release from a single MANiAC in phosphate buffered saline, pH 7.4.

## References

[1] Sitti M., Ceylan H., Hu W., Giltinan J., Turan M., Yim S., Diller E. (2015). Biomedical Applications of Untethered Mobile Milli/Microrobots. Proc. IEEE.

[2] Li J., Esteban-Fernandez de Ávila B., Gao W., Zhang L., Wang J. (2017). Micro/nanorobots for biomedicine: Delivery, surgery, sensing, and detoxification. Sci. Robot..

[3] Guix M., Weiz S.M., Schmidt O.G., Medina-Sanchez M. (2018). Self-Propelled Micro/Nanoparticle Motors. Part. Part. Syst. Charact..

[4] Jing W., Pagano N., Cappelleri D.J. (2013). A novel micro-scale magnetic tumbling microrobot. J. Micro-Bio Robot..

[5] Jing W., Cappelleri D. (2014). A Magnetic Microrobot with *in situ* Force Sensing Capabilities. Robotics.

[6] Jing W., Cappelleri D.J. Incorporating in situ force sensing capabilities in a magnetic microrobot. Proceedings of the IEEE/RSJ International Conference on Intelligent Robots and Systems.

[7] Guix M., An Z., Wang J., Johnson B., Cappelleri D.J. Vision-based micro-force sensing mobile microrobots for intelligent micromanipulation. Proceedings of the IEEE International Conference on Robotics, Manipulation, and Automation at Small Scales (MARSS).

[8] Bi C., Guix M., Johnson B.V., Jing W., Cappelleri D.J. (2018). Design of Microscale Magnetic Tumbling Robots for Locomotion in Multiple Environments and Complex Terrains. Micromachines.

[9] Jing W., Cappelleri D.J. (2015). Micro-force sensing mobile microrobots. Proc. SPIE 9494, Next-Generation Robotics II; and Machine Intelligence and Bio-inspired Computation: Theory and Applications IX.

[10] Aguero L., Zaldivar-Silva D., Pena L., Dias M. (2017). Alginate microparticles as oral colon drug delivery device: A review. Carbohydr. Polym..

[11] Lopes M., Abrahim B., Veiga F., Seiça R., Cabral L.M., Arnaud P., Andrade J.C., Ribeiro A.J. (2017). Preparation methods and applications behind alginate-based particles. Expert Opin. Drug Deliv..

[12] Paques J.P., van der Linden E., van Rijn C.J.M., Sagis L.M.C. (2014). Preparation methods of alginate nanoparticles. Adv. Colloid Interface Sci..

[13] Degen P., Zwar E., Schulz I., Rehage H. (2015). Magneto-responsive alginate capsules. J. Phys. Condens. Matter.

[14] Calafiore R., Basta G., Luca G., Lemmi A., Racanicchi L., Mancuso F., Montanucci M.P., Brunetti P. (2006). Standard Technical Procedures for Microencapsulation of Human Islets for Graft into Nonimmunosuppressed Patients With Type 1 Diabetes Mellitus. Transplant. Proc..

[15] Gonzalez-Pujana A., Orive G., Pedraz J.L., Santos-Vizcaino E., Hernandez R.M., Rehn B., Moradali M. (2018). Alginate Microcapsules for Drug Delivery. Alginates and Their Biomedical Applications.

[16] Calafiore R., Basta G., Luca G., Lemmi A., Pia Montanucci M., Calabrese G., Racanicchi L., Mancuso F., Brunetti P. (2006). Microencapsulated Pancreatic Islet Allograſts into Nonimmunosuppressed Patients with Type 1 Diabetes: First Two Cases. Diabetes Care.

[17] Tuch B.E., Keogh G.W., Williams L.J., Wu W., Foster J., Vaithilingam V., Phlips R. (2009). Safety and Viability of Microencapsulated Human Islets Transplated Into Diabetic Humans. Diabetes Care.

[18] Basta G., Montanucci P., Luca G., Boselli C., Noya G., Barbaro B., Qi M., Kinzer K.P., Oberholzer J., Calafiore R. (2011). Long-Term Metabolic and Immunological Follow-Up of Nonimmunosuppressed Patients with Type 1 Diabetes Treated with Microencapsulated Islet Allografts: Four cases. Diabetes Care.

[19] Ali J., Cheang U.K., Liu Y., Kim H., Rogowski L., Sheckman S., Patel P., Sun W., Kim M.J. (2016). Fabrication and magnetic control of alginate-based rolling microrobots. AIP Adv..

[20] Fusco S., Sakar M.S., Kennedy S., Peters C., Bottani R., Starsich F., Mao A., Sotiriou G.A., Pané S., Pratsinis S.E. (2014). An Integrated Microrobotic Platform for On-Demand, Targeted Therapeutic Interventions. Adv. Mater..

[21] Paxton W.F., Kistler K.C., Olmeda C.C., Sen A., Angelo S.K.S., Cao Y., Mallouk T.E., Lammert P.E., Crespi V.H. (2004). Catalytic Nanomotors: Autonomous Movement of Stripted Nanorods. J. Am. Chem. Soc..

[22] Xu T., Yu J., Yan X., Choi H., Zhang L. (2015). Magnetic Actuation Based Motion Control of Microrobots: An Overview. Micromachines.

[23] Wang W., Castro L.A., Hoyos M., Mallouk T.E. (2012). Autonomous Motion of Metallic Microrods Propelled by Ultrasound. ACS Nano.

[24] Ahmed S., Wang W., Mair L.O., Fraleigh R.D., Li S., Castro L.A., Hoyos M., Huang T.J., Mallouk T.E. (2013). Steering Acoustically Propelled Nanowire Motors toward Cells in a Biologically Compatible Environment Using Magnetic Fields. Langmuir.

[25] Wang W., Li S., Mair L., Ahmed S., Huang T.J., Mallouk T.E. (2014). Acoustic Propulsion of Nanorod Motors Inside Living Cells. Angew. Chem. Int. Ed.

[26] Balk A.L., Mair L.O., Mathai P.P., Patrone P.N., Wang W., Ahmed S., Mallouk T.E., Liddle J.A., Stavis S.M. (2014). Kilohertz Rotation of Nanorods Propelled by Ultrasound, Traced by Microvortex Advection of Nanoparticles. ACS Nano.

[27] Magdanz V., Medina-Sanchez M., Chen Y., Guix M., Schmidt O.G. (2015). How to improve spermbot performance. Adv. Funct. Mater..

[28] Schwarz L., Medina-Sánchez M., Schmidt O.G. (2017). Hybrid BioMicromotors. Appl. Phys. Rev..

[29] Wang W., Duan W., Zhang Z., Sun M., Sen A., Mallouk T.E. (2015). A tale of two forces: Simultaneous chemical and acoustic propulsion of bimetallic micromotors. Chem. Commun..

[30] Chautems C., Zeydan B., Charreyron S., Chatzipirpiridis G., Pané S., Nelson B.J. (2017). Magnetically powered microrobots: A medical revolution underway?. Eur. J. Cardiothorac. Surg..

[31] Alexiou C., Arnold W., Klein R.J., Parak F.G., Hulin P., Bergemann C., Erhardt W., Wagenpfeil S., Lubbe A.S. (2000). Locoregional Cancer Treatment with Magnetic Drug Targeting. Cancer Res..

[32] Lubbe A.S., Alexiou C., Bergemann C. (2001). Clinical applications of magnetic drug targeting. J. Surg. Res..

[33] Mair L.O., Superfine R. (2014). Single particle tracking reveals biphasic during nanorod magnetophoresis through extracellular matrix. Soft Matter.

[34] Shapiro B., Dormer K.J., Rutel I.B. (2010). A two-magnet system to push therapeutic nanoparticles. AIP Conf. Proc..

[35] Nacev A., Weinberg I.N., Stepanov P.Y., Kupfer S., Mair L.O., Urdaneta M.G., Shimoji M., Fricke S.T., Shapiro B. (2015). Dynamic Inversion Enables External Magnets To Concentrate Ferromagnetic Rods to a Central Target. Nano Lett..

[36] Tierno P., Johansen T.H., Fischer T.M. (2007). Magnetically driven colloidal microstirrer. J. Phys. Chem. B.

[37] Tierno P., Golestanian R., Pagonabarraga I., Sagués F. (2008). Controlled swimming in confined fluids of magnetically actuated colloidal rotors. Phys. Rev. Lett..

[38] Güell O., Sagues F., Tierno P. (2011). Magnetically driven Janus micro-ellipsoids realized via asymmetric gathering of the magnetic charge. Adv. Mater..

[39] Mair L.O., Evans B., Hall A.R., Carpenter J., Shields A., Ford K., Millard M., Superfine R. (2011). Highly controllable near-surface swimming of magnetic Janus nanorods: Application to payload capture and manipulation. J. Phys. Appl. Phys..

[40] Li T., Zhang A., Shao G., Wei M., Guo B., Zhang G., Li L., Wang W. (2018). Janus Microdimer Surface Walkers Propelled by Oscillating Magnetic Fields. Adv. Funt. Mater..

[41] Jiang G.-L., Guu Y.-H., Lu C.-L., Li P.-N., Shen H.-M., Lee L.-S., Yeh J.A. (2010). Development of rolling magnetic microrobots. J. Micromech. Microeng..

[42] Hou M.T., Shen H., Jiang G., Lu C., Hsu I., Yeh J.A. (2010). A rolling locomotion method for untethered magnetic microrobots. Appl. Phys. Lett..

[43] Sing C.E., Schmid L., Schneider M.F., Franke T., Alexander-Katz A. (2010). Controlled surface-induced flows from the motion of self-assembled colloidal walkers. Proc. Natl. Acad. Sci. USA.

[44] Zhang L., Petit T., Lu Y., Kratochvil B.E., Peyer K.E., Pei R., Lou J., Nelson B.J. (2010). Controlled propulsion and cargo transport of rotating nickel nanowires near a patterned solid surface. ACS Nano.

[45] Gabayno J.L.F., Liu D., Chang M., Lin Y. (2015). Controlled manipulation of Fe3O4 nanoparticles in an oscillating magnetic field for fast ablation of microchannel occlusion. Nanoscale.

[46] Mair L.O., Nacev A., Hilaman R., Stepanov P.Y., Chowdhury S., Jafari S., Hausfeld J., Karlsson A.J., Shirtliff M.E., Shapiro B. (2017). Biofilm disruption with rotating microrods enhances antimicrobial efficacy. J. Magn. Magn. Mater..

[47] Karle M., Wöhrle J., Miwa J., Paust N., Roth G., Zengerle R., Stetten F., Von Stetten F. (2010). Controlled counter-flow motion of magnetic bead chains rolling along microchannels. Microfluid. Nanofluid..

[48] Tung H.-W., Peyer K.E., Sargent D.F., Nelson B.J. (2013). Noncontact manipulation using a transversely magnetized rolling robot. Appl. Phys. Lett..

[49] Petit T., Zhang L., Peyer K.E., Kratochvil B.E., Nelson B.J. (2012). Selective trapping and manipulation of microscale objects using mobile microvortices. Nano Lett..

[50] Vach P.J., Fratzl P., Klumpp S., Faivre D. (2015). Fast Magnetic Micropropellers with Random Shapes. Nano Lett..

[51] Mair L.O., Evans B.A., Nacev A., Stepanov P.Y., Hilaman R., Chowdhury S., Jafari S., Wang W., Shapiro B., Weinberg I.N. (2017). Kilohertz Rotation of Nanorods Propelled by Ultrasound, Traced by Microvortex Advection of Nanoparticles. Nanoscale.

[52] Jing W., Chen X., Lyttle S., Fu Z., Shi Y., Cappelleri D.J. A magnetic thin film microrobot with two operating modes. Proceedings of the 2011 IEEE International Conference on Robotics and Automation.

[53] Joshi A., Solanki S., Chaudhari R., Bahadur D., Aslam M., Srivastava R. (2011). Multifunctional alginate microspheres for biosensing, drug delivery and magnetic resonance imaging. Acta Biomater..

[54] Shen F., Poncet-Legrand C., Somers S., Slade A., Yip C., Duft A.M., Winnik F.M., Chang P.L. (2003). Properties of a novel magnetized alginate for magnetic resonance imaging. Biotechnol. Bioeng..

[55] Garcia A.R., Lacko C., Snyder C., Bohórquez A.C., Schmidt C.E., Rinaldi C. (2017). Processing-size correlations in the preparation of magnetic alginate microspheres through emulsification and ionic crosslinking. Colloids Surf. Physicochem. Eng. Asp..

[56] Degen P., Leick S., Siedenbiedel F., Rehage H. (2012). Magnetic switchable alginate beads. Colloid Polym. Sci..

[57] Brulé S., Levy M., Wilhelm C., Letourneur D., Gazeau F., Ménager C., Le Visage C. (2011). Doxorubicin release triggered by alginate embedded magnetic nanoheaters: A combined therapy. Adv. Mater..

[58] Alshehri A.M., Wilson O.C., Dahal B., Philip J., Luo X., Raub C.B. (2017). Magnetic nanoparticle-loaded alginate beads for local micro-actuation of in vitro tissue constructs. Colloids Surf. B Biointerfaces.

[59] Barnett B.P., Arepally A., Karmarkar P.V., Qian D., Gilson W.D., Walczak P., Howland V., Lawler L., Lauzon C., Stuber M. (2007). Magnetic resonance–guided, real-time targeted delivery and imaging of magnetocapsules immunoprotecting pancreatic islet cells. Nat. Med..

[60] Barnett B.P., Arepally A., Stuber M., Arifin D.R., Kraitchman D.L., Bulte J.W.M. (2011). Synthesis of magnetic resonance-, X-ray- and ultrasound-visible alginate microcapsules for immunoisolation and noninvasive imaging of cellular therapeutics. Nat. Protoc..

[61] Arifin D.R., Valdeig S., Anders R.A., Bulte J.W.M., Weiss C.R. (2016). Magnetoencapsulated human islets xenotransplanted into swine: A comparison of different transplantation sites. Xenotransplantation.

[62] Kim J., Arifin D.R., Muja N., Kim T., Gilad A.A., Kim H., Arepally A., Hyeon T., Bulte J.W.M. (2011). Multifunctional Capsule-in-Capsules for Immunoprotection and Trimodal Imaging. Angew. Chem. Int. Ed Engl..

[63] Mills P.H., Hitchens T.K., Foley L.M., Link T., Ye Q., Weiss C.R., Thompson J.D., Gilson W.D., Arepally A., Melick J.A. (2012). Automated detection and characterization of SPIO-labeled cells and capsules using magnetic field perturbations. Magn. Reson. Med..

[64] Finotelli P.V., Da Silva D., Sola-Penna M., Rossi A.M., Farina M., Andrade L.R., Takeuchi A.Y., Rocha-Leão M.H. (2010). Microcapsules of alginate/chitosan containing magnetic nanoparticles for controlled release of insulin. Colloids Surf. B Biointerfaces.

[65] Wang G.-J., Lin Y.-C., Li C.-W., Hsueh C.-C., Hsu S.-H., Hung H.-S. (2009). Fabrication of orderly nanostructured PLGA scaffolds using anodic aluminum oxide templates. Biomed. Microdevices.

[66] Miyashita S., Guitron S., Yoshida K., Li S., Damian D.D., Rus D. Ingestible, Controllable, and Degradable Origami Robot for Patching Stomach Wounds. Proceedings of the 2016 IEEE International Conference on Robotics and Automation (ICRA).

[67] Martin C.R. (1994). Nanomaterials: A Membrane-Based Synthetic Approach. Science.

[68] Qin J., Nogués J., Mikhaylova M., Roig A., Muñoz J.S., Muhammed M. (2005). Differences in the magnetic properties of Co, Fe, and Ni 250-300 nm wide nanowires electrodeposited in amorphous anodized alumina templates. Chem. Mater..

[69] Hurst S.J., Payne E.K., Qin L., Mirkin C.A. (2006). Multisegmented one-dimensional nanorods prepared by hard-template synthetic methods. Angew. Chem. Int. Ed..

[70] Paredes-Juarez G.A., De Haan B.J., Faas M.M., De Vos P. (2013). The role of pathogen-associated molecular patterns in inflammatory responses against alginate based microcapsules. J. Control. Release.

[71] Paredes-Juarez G.A., de Vos P., Bulte J.W.M. (2017). Recent progress in the use and tracking of transplanted islets as a personalized treatment for type 1 diabetes. Expert Rev. Precis. Med. Drug Dev..

[72] Schuerle S., Erni S., Flink M., Kratochvil B.E., Nelson B.J. (2013). Three-Dimensional Magnetic Manipulation of Micro- and Nanostructures for Applications in Life Sciences. IEEE Trans. Magn..

[73] Taylor R.M. Video Spot Tracker software, Version 6.04. http://cismm.web.unc.edu/software/.

[74] Keshoju K., Xing H., Sun L. (2007). Magnetic field driven nanowire rotation in suspension. Appl. Phys. Lett..

[75] Stoner E.C., Wohlfarth E.P. (1948). A Mechanism of Magnetic Hystersis in Heterogeneous Alloys. Philos. Trans. R. Soc. Lond. Ser. Math. Phys. Sci..

[76] Balk A.L., Mair L.O., Guo F., Hangarter C., Mathai P.P., Mcmichael R.D., Stavis S.M., Unguris J. (2015). Quantitative magnetometry of ferromagnetic nanorods by microfluidic analytical magnetophoresis. J. Appl. Phys..

[77] Mair L., Ford K., Alam M.R., Kole R., Fisher M., Superfine R. (2009). Size-Uniform 200 nm Particles: Fabrication and Application to Magnetofection. J. Biomed. Nanotechnol..

[78] Goldman A.J., Cox R.G., Brenner H. (1967). Slow Viscous Motion of a Sphere Parallel to a Plane Wall I: Motion through a Quiscent Fluid. Chem. Eng. Sci..

[79] Feuillebois F., Gensdarmes F., Mana Z., Ricciardi L., Monier C., Le Meur G., Reynaud C., Rabaud M. (2016). Three-dimensional motion of particles in a shear flow near a rough wall. J. Aerosol Sci..

[80] Hsu R., Ganatos P. (1994). Gravitational and zero-drag motion of a spheroid adjacent to an inclined plane at low-Reynolds Number. J. Fluid Mech..

[81] Chaoui M., Feuillebois F. (2003). Creeping Flow Around a Sphere in a Shear Flow Close to a Wall. Q. J. Mech. Appl. Math..

[82] Kuo C.-W., Lai J.-J., Wei K.H., Chen P. (2007). Studies of Surface-Modified Gold Nanowires Inside Living Cells. Adv. Funct. Mater..

[83] Tung H.W., Sargent D.F., Nelson B.J. (2014). Protein crystal harvesting using the RodBot: A wireless mobile microrobot. J. Appl. Crystallogr..

[84] Jain D., Bar-Shalom D. (2014). Alginate drug delivery systems: Application in context of pharmaceutical and biomedical research. Drug Dev. Ind. Pharm..

[85] Esteban-Fernández de Ávila B., Angsantikul P., Li J., Lopez-Ramirez M.A., Ramírez-Herrera D.E., Thamphiwatana S., Chen C., Delezuk J., Samakapiruk R., Ramez V. (2017). Micromotor-enabled active drug delivery for in vivo treatment of stomach infection. Nat. Commun..

[86] Li J., Thamphiwatana S., Liu W., Esteban-Fernández de Ávila B., Angsantikul P., Sandraz E., Wang J., Xu T., Soto F., Ramez V. (2016). An Enteric Micromotor Can Selectively Position and Spontaneously Propel in the Gastrointestinal Tract. ACS Nano.

